# Impact of Arteriovenous fistula creation on estimated glomerular filtration rate decline in Predialysis patients

**DOI:** 10.1186/s12882-019-1607-4

**Published:** 2019-11-25

**Authors:** Valérie Bénard, Maude Pichette, Jean-Philippe Lafrance, Naoual Elftouh, Vincent Pichette, Louis-Philippe Laurin, Annie-Claire Nadeau-Fredette

**Affiliations:** 1 Division of Nephrology Hôpital Maisonneuve-Rosemont, 5415, l’Assomption blvd., Quebec, Montreal H1T 2M4 Canada; 2 Research Center, Hôpital Maisonneuve-Rosemont, 5415, l’Assomption blvd., Quebec, Montreal H1T 2M4 Canada; 3 Department of pharmacology and physiology, Université de Montréal, Montreal, Quebec, Canada

**Keywords:** Arteriovenous fistula, Chronic kidney disease, Estimated glomerular filtration rate, Predialysis, Prevention, Louis-Philippe Laurin and Annie-Claire Nadeau-Fredette are contributed equally to this work.

## Abstract

**Background:**

Arteriovenous fistula (AVF) is the vascular access of choice for patients on hemodialysis. Recent evidence suggests that AVF creation may slow estimated glomerular filtration rate (eGFR) decline. The study objective was to assess the impact of the AVF creation on eGFR decline, after controlling for key confounding factors.

**Methods:**

This retrospective cohort study included adult patients followed in a single-center predialysis clinic between 1999 and 2016. Patients with a patent AVF were followed up to 2 years pre- and post-AVF creation. Estimated GFR trajectory was reported using linear mixed models adjusted for demographic characteristics, comorbidities and use of renin-angiotensin-aldosterone blockade.

**Results:**

A total of 146 patients were studied with a median age 68.7 (60.5–75.4) years and a median eGFR at time of AVF creation of 12.8 (11.3–13.9) mL/min/1.73m^2^. The crude annual eGFR decline rates were − 3.60 ± 4.00 mL/min/1.73 m^2^ pre- and − 2.28 ± 3.56 mL/min/1.73 m^2^ post-AVF, resulting in a mean difference of 1.28 mL/min/1.73 m^2^ (95% CI 0.49, 2.07). In a mixed effect linear regression model, monthly eGFR decline was − 0.63 (95% CI -0.81, − 0.46; *p* <  0.001) mL/min/1.73m^2^/month. The period after AVF creation was associated with a relatively higher eGFR (β 0.94, 95% CI 0.61–1.26, *p* <  0.001). There was a significant association between follow-up time and the period pre/post AVF (β 0.19, 95% CI 0.16, 0.22; p <  0.001) such that eGFR decline was more attenuated each month after AVF creation.

**Conclusions:**

In this cohort, AVF creation was associated with a significant reduction of eGFR decline. Further prospective studies are needed to confirm this association.

## Background

Chronic kidney disease (CKD) is a global health problem and end-stage kidney disease (ESKD) prevalence has increased over the past decade [1, 2]. In 2014, nearly 90% of all newly diagnosed ESKD patients began renal replacement therapy with in-center hemodialysis in United States (U.S.) [2]. In Canada, the incidence of ESKD doubled from 1994 to 2014, mostly due to an increase in the elderly population [3]. Hemodialysis was the initial treatment for more than 75% of Canadian patients [3]. Hospitalisations and cardiovascular mortality are strongly linked to estimated glomerular filtration rate (eGFR) decline [2]. In addition to being expensive for the health care system [3–5] and associated with adverse events [2, 6–8], hemodialysis requires important adaptations from patients and their caregivers [7, 9]. Hence, delay of hemodialysis initiation is favoured for medical, psychosocial and financial reasons.

Recently, authors raised the hypothesis that arteriovenous fistula (AVF) creation might slow CKD progression [10, 11] and consequently delay hemodialysis. Overall, only few interventions have been shown to delay CKD progression including blood pressure control and proteinuria reduction though use of renin-angiotensin-aldosterone system (RAAS) blockers, treatment of metabolic acidosis and glycemic control [12]. Hence, the identification of a potentially new target to slow CKD progression is of the greatest interest, especially knowing that AVF is considered the best vascular access for chronic hemodialysis due to its superior durability and lower risk of adverse events compared to central venous catheter (CVC) or arteriovenous graft (AVG) [13] .

This study aimed to assess the association between AVF creation and eGFR decline in CKD patients. It was postulated that eGFR decline would be slower after AVF creation even when taking into account potential confounders.

## Methods

### Study design

This retrospective cohort study included CKD patients attending a tertiary care academic hospital the pre-dialysis clinic, between 1999 and 2016. The Institutional Research Ethics Board approved the study.

### Subjects

Patients were eligible if they (i) were aged 18 years and older, (ii) were followed in the pre-dialysis clinic before dialysis initiation, (iii) had a nephrologist-confirmed patent native AVF and (iv) two documented eGFR values both in the 6 months pre- and post-AVF. Patients with a previous renal transplantation and patients with any other hemodialysis vascular access (CVC, AVG) during the 6 months pre- and post-AVF were excluded.

### Measurements, covariates and outcomes

The medical charts of all eligible patients were identified by the medical archivist based on surgically created AVF for dialysis [Canadian Classification of Health Interventions (CCI) code 1.KY.76.LA and International Statistical Classification of Diseases and Related Health Problems, 10th Revision, Canada (ICD-10-CA) code Z99.2]. Patient’s demographics at AVF creation [age, sex, ethnicity, body mass index (BMI)], comorbidities, smoking status (active or not) and primary kidney disease were documented. Values of eGFR at study initiation, AVF creation and end of follow-up were recorded. In addition, use of RAAS blockers and loop diuretics (furosemide) pre-AVF creation were documented. Hospitalisations pre-AVF creation were also taken into account. End of patient follow-up included initiation of hemodialysis, end of follow-up (2 years post-AVF or November 2016), transplantation, and death, whichever occurred first. Patent AVF was defined as documented presence of an AVF thrill and/or use of the AVF at time of RRT initiation. The study primary outcome was the adjusted change of eGFR slope after AVF creation compared to before access creation.

### Statistical analysis

Characteristics of the cohort were summarized using the mean and standard deviation (SD) or median and interquartile range (IQR) for continuous variables normally and non-normally distributed, respectively, and proportions for categorical variables. Monthly eGFR values were calculated using the CKD-EPI formula [14]. The crude within-patient eGFR variation pre- and post-AVF was calculated using paired t-test. Within patient adjusted eGFR trajectory pre- and post-AVF were analyzed by multivariate linear mixed model with time zero being AVF creation date [15].. Covariates included in the model were based on pre-specified forced-in potential confounding factors (age, sex, race, diabetes status) with the additional inclusion of covariates with a *p*-value < 0.2 in a complete model including all variables listed in Table 1. Two-level interactions between follow-up time and each of these variables was also added to the complete model to account for their effect on eGFR change though time. Covariates with a *p*-value ≥0.2 were eliminated from the final model only if their exclusion did not significantly change other associations. A first sensitivity analysis was performed in order to alleviate retention bias by excluding patients with follow-up time after AVF exceeding 2 years. The second sensitivity analysis aimed to assess the potential effect of RAAS blocker change by excluding patients who started or stopped of RAAS blocker after AVF creation. All tests were two-sided with *p* < 0.05 considered statistically significant. Analyses were performed using SAS software (version 9.4, SAS Institute Inc. Cary, U.S.).

**Table 1:** Baseline characteristics

Variable	*n* = 146
Age (years)	69 (61–75)
Female sex	67 (46)
BMI^a^	30 (27–34)
eGFR (mL/min/1.73 m^2^)	
First eGFR during study period	18 (15–22)
eGFR at AVF creation	13 (11–14)
Race	
Black	7 (5)
White	128 (88)
Other	11 (8)
Comorbidities	
Hypertension	142 (97)
Diabetes	89 (61)
Cardiovascular disease	67 (46)
Chronic obstructive pulmonary disease	24 (16)
Heart failure	9 (6)
Active smoking	24 (16)
Primary kidney disease^b^	
Benign nephrosclerosis	54 (38)
Diabetic nephropathy	51 (35)
Glomerulonephritis	25 (17)
Others	14 (10)
Furosemide use pre-AVF	103 (70)
RAAS blockade use pre-AVF	111 (76)
Patients with ≥1 hospitalisation pre-AVF	56 (38)

Results are presented as number (proportion) or median (interquartile range)

*BMI* Body mass index, *eGFR* estimated glomerular filtration rate, *AVF* arteriovenous fistula, *RAAS* Renin angiotensin aldosterone system

^a^BMI, *n* = 108; ^b^ primary kidney disease, *n* = 144;

## Results

A total of 146 eligible patients were included in the analysis. Baseline characteristics of the study cohort are displayed in Table 1. The median eGFR at AVF creation was 12.8 (IQR 11.3–14.0) mL/min/1.73 m^2^. The median (IQR) observation periods pre- and post-AVF creation was 22 (18–23) months and 14 (9–21) months, respectively while median number of eGFR observations before and after AVF creation was 9 (IQR 7–11) and 7 (IQR 5–10). Median eGFR at hemodialysis initiation (*n* = 78) was 8.2 (IQR 6.9–9.9) mL/min/1.73 m^2^and the median time between AVF creation and dialysis start among these patients was 332 (246–472) days. (Table 2) Overall, there was no significant difference in mean systolic blood pressure (BP) before and after AVF creation (145.9 ± 18.9 vs. 146.6 ± 17.2 mmHg, *p* = 0.49). Mean diastolic BP was however lower after AVF creation (71.7 ± 8.6) compared to before AVF creation (75.1 ± 10.4, *p* < 0.001). Furthermore, there was no statistically significant difference in weight before and after AVF creation (82.1 ± 19.2 kg vs. 81.0 ± 19.3 kg, *p* = 0.92). Only 25 patients (17%) had a cardiac ultrasound before and after AVF creation. Of these, six patients had a lower left ventricular ejection fraction (LVEF) after AVF creation compared to before, one patient had an improved LVEF and the other 18 patients had a stable LVEF.

**Table 2:** Estimated glomerular filtration rate at end of follow-up

End of follow-up causes	n (%)	Median	Interquartile range
Hemodialysis initiation	78 (53)	8.2	6.9–9.9
Death	4 (3)	9.0	6.5–17.2
Transplantation	6 (4)	10.7	9.3–13.5
End of study period	58 (40)	12.3	8.8–15.1

### Crude eGFR decline

**Fig. 1:**
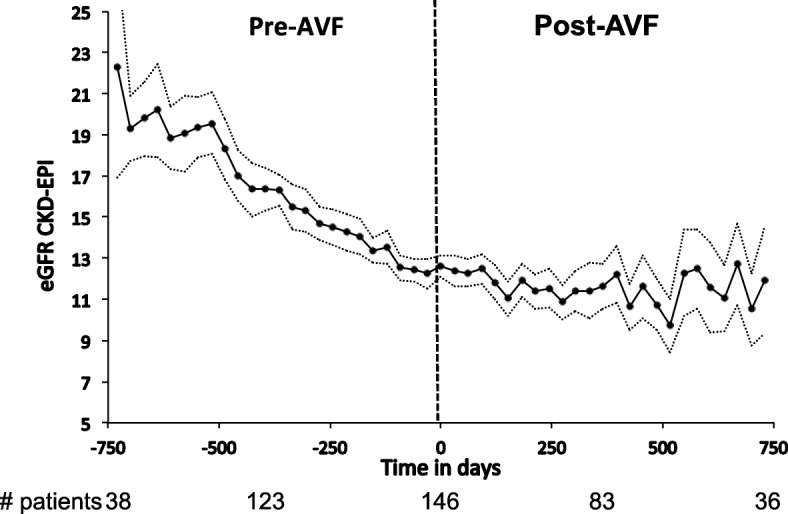
Crude annual eGFR decline pre- and post-AVF creation, displays the eGFR in mL/min/1.73 m^2^, calculated using the CKD-EPI formula, against the time in days before and after AVF creation, the latter represented by time 0. The estimates are depicted by the bold solid line with the 95% confidence interval in dotted lines. The crude annual eGFR slope was −3.60 ± 4.00 ml/min/1.73m^2^ pre-AVF and of −2.28 ± 3.56 ml/min/1.73 m^2^ post-AVF (mean difference 1.28 ml/min/1.73m^2^; 95% CI: 0.49, 2.07; *p* = 0.002)

The crude eGFR decline significantly slowed after AVF creation with a mean annual eGFR decline rate of − 3.60 ± 4.00 mL/min/1.73 m^2^ pre-AVF and of − 2.28 ± 3.56 mL/min/1.73 m^2^ post-AVF (mean difference 1.28 mL/min/1.73 m^2^; 95% CI: 0.49, 2.07; *p* = 0.002) (Fig. 1). The crude difference in eGFR decline rate reached statistical significance at 12 months pre- and post-AVF creation (Additional file 1: Figure S1 A, B, C).

### Predicted eGFR trajectory

In an unadjusted model, the period after AVF creation was associated with a slower eGFR decline (follow-up time * AVF period β 0.19 95% CI 0.16, 0.22). This association was preserved when adjusting for key confounding factors, with a statistically significant association between AVF creation and follow-up time, such that predicted monthly eGFR decline (β − 0.63, 95% CI -0.81, − 0.46, per month) slowed by 0.19 (95% CI 0.16, 0.22) mL/min/1.73 m^2^ each month after AVF creation (Table 3). Practically, this means predicted eGFR decreased by only 0.44 mL/min/1.73 m^2^ each month after AVF creation compared to 0.63 mL/min/1.73 m^2^ each month before AVF creation. The period after AVF creation was globally associated with a higher predicted eGFR (β0.94, 95% CI 0.61–1.26), although this effect was modest when taking into account the monthly predicted eGFR decline reported above.

**Table 3:** Determinants of the eGFR - Adjusted mixed effect linear regression

Covariates	β Estimate (95% CI)	*P*-value
Follow-up time (months)	−0.63 (− 0.81, − 0.46)	< 0.001
Period after AVF (ref. before)	0.94 (0.61, 1.26)	< 0.001
Follow-up time * period after AVF (ref. before)	0.19 (0.16, 0.22)	< 0.001
Female	−0.69 (−1.48, 0.11)	0.09
Age (per 5-year increase)	0.03 (−0.13, 0.18)	0.73
Follow-up time * Age	0.02 (0.01–0.04)	0.001
Black race (ref. White/other)	2.73 (0.89, 4.57)	0.004
Follow-up time * Black race	−0.20 (−0.35, − 0.05)	0.01
RAAS blockade use	1.82 (0.89, 2.75)	< 0.001
Diabetes	0.87 (0.03, 1.71)	0.04

*AVF* arteriovenous fistula, *RAAS* renin angiotensin aldosterone system

Predictors of higher eGFR at AVF creation (time = 0) included age, Black race, diabetes and RAAS blockade use. In contrast, male sex was associated with lower eGFR. Furthermore, eGFR decline was also attenuated in older patients (follow-up time * age β 0.02, 95% CI 0.01, 0.04) and accentuated in Black patients (follow-up time * Black race β − 0.20, 95% CI -0.35, − 0.05).

### Sensitivity analysis

A first sensitivity analysis was performed to exclude patients who were followed more than 2 years in the predialysis clinic after AVF creation without requiring dialysis initiation, assuming these patients might have a more stable kidney function favorably altering the association between AVF creation and eGFR decline. Overall, the results remained consistent when excluding these 33 patients, with a statistically significant attenuation in monthly eGFR decline after AVF creation (β0.17, 95% CI 0.13. 0.22) (Additional file 1: Table S1, Additional file 1: Table S2 and Table S3).

A second sensitivity model was undertaken excluding patients with a change of RAAS blockade status, assuming this intervention might influence the reported association. Overall, 19 patients stopped RAAS blockade after AVF creation while 12 patients initiated RAAS blockade. The association between AVF and eGFR decline remained consistent in this sensitivity model (β0.16, 95% CI 0.13. 0.20) for the ‘time*period after AVF’ variable (Additional file 1: Table S4).

## Discussion

In this cohort of adult CKD patients followed in a predialysis clinic, crude eGFR decline slowed by 1.28 mL/min/1.73 m^2^ per year during the period after AVF creation compared to before. The predicted eGFR was lower after AVF creation and this association persisted after controlling for key confounding factors. Furthermore, the protective association between AVF and eGFR was magnified each month with an additional attenuation of eGFR decline of 0.19 mL/min/1.73 m^2^ per month. This monthly slowing of eGFR decline was clinically important, translating in kidney function preservation of 2.28 mL/min/1.73 m^2^ per year. This study is the first to show an adjusted time-dependent effect of AVF creation where the association between AVF and slowing of eGFR was more pronounced each month after AVF creation.

These findings are globally consistent with two recent studies (12,13). Golper and colleagues reported pronounced changes in crude eGFR slopes before (− 5.90 mL/min/1.73 m^2^) and after (− 0.46 mL/min/1.73 m^2^; *p* < 0.001) AVF creation in 123 CKD patients from a single center [10]. The marked attenuation in eGFR decline after AVF creation in their study could be related to several factors including higher eGFR at time of AVF creation (16.9 vs. 12.8 mL/min/1.73 m^2^ in the present study), differences in populations for which the lack of detailed data on demographics, comorbidities and medication in Golper’s study prevented effective comparison and differences in practice patterns. Overall, the authors did not report adjusted analysis, which greatly limited the extent of their results. Sumida and colleagues reported a similar association with a crude eGFR slope of − 5.60 mL/min/1.73 m^2^ pre-AVF/AVG creation and − 4.10 mL/min/1.73 m^2^ post-AVF/AVG, (*p* < 0.001), irrespectively of the access maturation status in a cohort including 3026 veterans [11]. This association remained significant in adjusted analyses. The authors also described eGFR slope changes in patients without AVF/AVG creation attempt. These patients had a surprisingly high eGFR decline rate of − 16.3 mL/min/1.73 m^2^ during the last 6 months before hemodialysis start (cut-off arbitrary defined) compared to the previous period (− 6.0 mL/min/1.73 m^2^), which may alter the generalizability of their results. Moreover, the study population included an overrepresentation of males (98%) and patients with heart failure (50%), which combined with and the lack of data on primary kidney disease, may limit the extent of the results [2]. Of note, eGFR at time of AVF/AVG creation, a critical data for vascular access, was not reported. Recently, the association between AVF and eGFR slope was further supported by a publication suggesting a negative impact of AVF closure on eGFR slope in kidney transplant recipients. This study included 345 patients among whom 114 had AVF ligature 1.8 ± 1.2 years after kidney transplantation [16]. In these patients, eGFR slope significantly deteriorated after AVF closure (− 1.9 mL/min/year) compared to before (0.47 mL/min/year, *p* = 0.03).

Several factors have been associated with an accelerated eGFR decline including young age, proteinuria, male sex, systolic hypertension and diabetes. In contrast, ACE inhibitor and ARB are known to slow eGFR decline [17, 18]. In the present study, black race was associated with steeper eGFR decline through time while increased age was associated with slower kidney function decline.

Different mechanisms have been proposed to explain the observed benefits of AVF creation on kidney function [10, 11]. First, a remote ischemic preconditioning (RIPC) at time of access creation may be implicated. Recent studies demonstrated that RIPC, induced by repeated cycle of inflating and deflating blood pressure cuffs, could enhance renal protection against ischemic injuries through various humoral, anti-inflammatory, anti-oxydant and anti-apoptotic effect [8, 11, 19–22]. Local limb ischemia induced by clamping of arteries and ligature of small arterioles during AVF creation could create acute changes similar to those observed with RIPC. Additionally, chronic subclinical ischemia could be induced by subclinical arterial steal, visualized as a retrograde flow in radial artery, reported to be present in more than 70% of patients with AVF [23]. Second, various hemodynamic changes are known to occur as early as 2 weeks post AVF creation and could support their favorable effect of eGFR slope. AVF enhances cardiac output through increased heart rate, cardiac contractility and venous return, and lowers systemic peripheral resistance and arterial rigidity through endothelial changes induced by increased wall stress [24, 25]. The enhanced cardiac output and lower systemic peripheral resistance both favor renal perfusion [24]. Since CKD progression has been associated with an elevation in arterial stiffness, which further deteriorates kidney function [26, 27], it is possible that AVF creation acts as a stabilizer of this negative loop. In this study, there was a statistically significant decrease in diastolic BP after AVF creation but no significant change in systolic BP. A more pronounced decrease in diastolic BP (− 3.9 mm HG) than systolic BP (− 1.7 mm HG) after AVF creation (compared to patients with CVC) was also reported in a propensity score matching study by Mathew and colleagues [28]. It is unsure how relevant the BP finding is in relation to changes in systemic peripheral resistance, especially considering that BP values were obtained with single-measure clinic observation, rather than home monitoring, repeated measures or central BP assessment. Furthermore, changes in all BP medication after AVF creation were not available, which might also influence BP readings. Of note, a similar analysis was performed with patients excluded from the study due to non-patency (*n* = 18). In this small group, there was no significant crude annual difference in eGFR decline before and after AVF creation (− 6.11 ± 12.37 mL/min/1.73 m^2^ pre-AVF and − 4.03 ± 4.45 mL/min/1.73 m^2^ post-AVF (mean difference 2.08 mL/min/1.73 m^2^; *p* = 0.78). Due to the small sample size, this analysis should be interpreted with great caution.

Alternatively, despite the biological plausibility described above, it is possible that the observed effect of AVF on eGFR was mediated by improved compliance to drug and medical follow-up or natural stabilization of the kidney disease. Of note, Lynch and colleagues showed that despite specific quality-improvement intervention after AVF creation, patients compliance to follow-up remained poor (only 22% of patients attended their second appointment) and AVF outcomes were unchanged in this group [29]. Hence, it is unlikely that the significant interaction between AVF and eGFR decline is solely related to an enhanced medical and behavioural patient compliance. These elements were however not assessed in the present study and should be evaluated in further work. Additionally, previous studies have reported faster kidney function decline before dialysis start than the eGFR slope reported in the present study, mitigating the assumption that the native kidney function may naturally stabilize before dialysis initiation [30, 31]. Nonetheless, considering the nature of the study data, a residual indication bias with referral for AVF after a steeper decline in eGFR and subsequent natural stabilization of the kidney function cannot be excluded.

AVF is considered the optimal vascular access due to its lower adverse events rate and longer durability [13]. Nevertheless, the U.S. and especially Canada rank poorly in terms of AVF use, even though the proportion of patients seen by a nephrologist 6 month before dialysis initiation is greater in Canada than in other countries [2, 32]. Confirmation of the positive impact of AVF on eGFR would justify greater efforts toward AVF planning, and potentially, recommendation for earlier AVF creation in predialysis patients. Currently, the thresholds considered appropriate to create an AVF are 12 months before estimated hemodialysis start or an eGFR of 15–20 mL/min/1.73 m^2^ [33].

Whether the beneficial effect of AVF on eGFR applies to all patients remain uncertain. In the current study, heart failure was not statistically significantly associated with eGFR decline, although only 6% of the patients had documented heart failure at baseline, which limited the analysis. The association between cardiac and renal function is well supported by knowledge detailing cardio-renal syndrome [34]. Considering the known relationship between AVF and cardiovascular events [35, 36] and the frequency of undiagnosed AVF-induced high output cardiac failure [37–39], AVF creation should be performed with caution in patients with severe heart failure. Upper arm AVFs have been associated with an increased risk of high-output cardiac failure due to higher vascular access flow compared to lower arm AVFs [24], which may be a preferable access in patients with heart failure. AVF is also known to worsen pulmonary hypertension and potentially precipitate right-sided heart failure [40]. In the present study, AVF location, baseline NYHA class and right versus left-sided heart failure were not taken into account, and only 6% of patients suffered from heart failure at baseline, limiting its generalizability in this population. Of note, in the subgroup of patients (*n* = 25) with sequential cardiac ultrasound before and after AVF creation, 6 patients had a lower LVEF after AVF placement compared to before. Due to the small sample size and potential indication bias for repeated ultrasound, this data should be interpreted with caution.

This study has several strengths including its adjustment for important potential confounders and the inclusion of a large predialysis cohort representative of the Canadian CKD population [3]. The results were consistent in a sensitivity model excluding patients with an extended predialysis follow-up. Ultimately, the beneficial association between AVF creation and eGFR decline rate found in the present study was consistent with previous studies and supported by plausible physiological mechanisms such as RIPC and hemodynamic variations [10, 11].

The study results must be viewed in the context of several limitations related to its single-center retrospective design. The observational study design impeded the information available on important factors such as therapeutic compliance and acute change in health status. Moreover, the study was not designed to assess potential AVF complications neither than patients more likely to benefit from AVF. The timing of AVF creation was left at the discretion of the nephrologist, who must take into account many factors in this decision, including uremic symptoms, patient’s age and the decline rate of renal function. While it cannot rule-out that a different timing of AVF creation may have modified the results, the steep decline in renal function prior to AVF underlines the appropriateness of the indication. Finally and importantly, the study cohort lacked a control group without functioning AVF, which could have provided further data on the potential causality underling the association between AVF and CKD progression.

## Conclusions

In conclusion, this study showed a significant decrease in eGFR slope after AVF creation in CKD patients followed in the pre-dialysis setting, irrespectively of medication, demographics and comorbidities. These results raise the question as to whether the optimal timing of AVF creation should be earlier in the course of severe CKD to potentially attenuate eGFR decline. Further prospective studies are indicated to evaluate with greater details the interaction between AVF creation and advanced CKD progression.

## Supplementary information

**Additional file 1: Table S1.** Baseline characteristics excluding patients with stable kidney function. **Table S2.** Estimated glomerular filtration rate at end of follow-up excluding patients with stable kidney function. **Table S3.** Determinants of the eGFR excluding patients with stable kidney disease- Adjusted mixed effect linear regression. **Table S4.** Determinants of the eGFR excluding patients with a change in RAAS blockade use - Adjusted mixed effect linear regression. **Figure S1.** Annual eGFR decline pre- and post-AVF creation over different observational periods.

## Data Availability

The dataset used and/or analyzed during the current study are available from the corresponding authors on reasonable request.

## A**bbreviations**

AVF: Arteriovenous fistula
AVG: Arteriovenous graft
BMI: Body mass index
BP: Blood pressure
CKD: Chronic kidney disease
CVC: Central venous catheter
eGFR: Estimated glomerular filtration rate
ESKD: End-stage kidney disease
LVEF: Left ventricular ejection fraction
RAAS: Renin-angiotensin-aldosterone system
RIPC: Remote ischemic preconditioning
