# Supplementary material for: Impact of Arteriovenous fistula creation on estimated glomerular filtration rate decline in Predialysis patients
Source: BMC Nephrol. 2019 Nov 25;20:420. doi: 10.1186/s12882-019-1607-4 (PMC6876290; doi:10.1186/s12882-019-1607-4)
Supplement: Supplementary file 1 — Additional file 1: Table S1. Baseline characteristics excluding patients with stable kidney function. Table S2. Estimated glomerular filtration rate at end of follow-up excluding patients with stable kidney function. Table S3. Determinants of the eGFR excluding patients with stable kidney disease- Adjusted mixed effect linear regression. Table S4. Determinants of the eGFR excluding patients with a change in RAAS blockade use - Adjusted mixed effect linear regression. Figure S1. Annual eGFR decline pre- and post-AVF creation over different observational periods. [file 12882_2019_1607_MOESM1_ESM.docx]

**SUPPLEMENTAL MATERIAL PDF**

Table S1. Baseline characteristics excluding patients with stable kidney function

| Variable | n=113 |
| --- | --- |
| Age (years) | 68 (60-75) |
| Sex (female) | 54 (48) |
| BMI (kg/m^2^) | 30 (26-34) |
| eGFR (mL/min/1.73 m^2^) |  |
| First eGFR | 17 (15-21) |
| eGFR at AVF | 12 (11-14) |
| Race |  |
| White | 97 (86) |
| African American | 6 (5) |
| Other | 10 (8) |
| Co-morbidities |  |
| Hypertension | 109 (97) |
| Diabetes | 67 (60) |
| Cardiac and vascular diseases | 49 (43) |
| Chronic obstructive pulmonary disease | 20 (18) |
| Heart failure | 6 (5) |
| Active smoking | 19 (17) |
| Primary kidney disease |  |
| Benign nephrosclerosis | 41 (37) |
| Diabetic nephropathy | 40 (36) |
| Glomerulonephritis | 21 (19) |
| Others | 10 (9) |
| Furosemide use | 82 (73) |
| Patients with ≥ 1 hospitalisation pre-AVF | 46 (41) |

*BMI: Body mass index, n=80, eGFR: estimated glomerular filtration rate

Results are presented as number (proportion) or median (interquartile range)

Table S2. Estimated glomerular filtration rate at end of follow-up excluding patients with stable kidney function

| **End of follow-up causes** | **n (%)** | **Median** | **IQR** |
| --- | --- | --- | --- |
| Hemodialysis initiation | 78 (69) | 8.2 | 6.9-9.9 |
| Death | 4 (4) | 9.0 | 6.5-17.2 |
| Transplantation | 6 (5) | 10.7 | 9.3-13.5 |
| End of study period | 25 (22) | 12.4 | 8.8-15.0 |

Table S3. Determinants of the eGFR excluding patients with stable kidney disease- Adjusted mixed effect linear regression

| **Covariates** | **β Estimate (95% CI)** | **P-value** |
| --- | --- | --- |
| Follow-up time (months) | -0.65 (-0.86, -0.44) | <0.0001 |
| Period after AVF (ref. before) | 0.86 (0.49, 1.22) | <0.0001 |
| Follow-up time * period after AVF | 0.17 (0.13, 0.22) | <0.0001 |
| Female | -0.27 (-1.08, 0.54) | 0.51 |
| Age (per 5-year increase ) | 0.06 (-0.08, 0.21) | 0.39 |
| Follow-up time * Age | 0.02 (0.01, 0.04) | 0.005 |
| Black race (ref. White/other) | 3.22 (1.47, 4.98) | 0.0004 |
| Follow-up time * Black race | -0.24 (-0.42, -0.06) | 0.008 |
| RAAS blockade use | 1.22 (0.31, 2.13) | 0.009 |
| Diabetes | 1.03 (0.17, 1.89) | 0.02 |

AVF: arteriovenous fistula, RAAS: renin angiotensin aldosterone system

Supplementary table S4. Determinants of the eGFR excluding patients with a change in RAAS blockade use - Adjusted mixed effect linear regression

| **Covariates** | **β Estimate (95% CI)** | **P-value** |
| --- | --- | --- |
| Follow-up time (months) | -0.61 (-0.79, -0.43) | <0.001 |
| Period after AVF (ref. before) | 0.78 (0.44, 1.13) | <0.001 |
| Follow-up time * period after AVF | 0.16 (0.13, 0.20) | <0.001 |
| Female | -1.02 (-1.87, -0.16) | 0.02 |
| Age (per 5-year increase ) | 0.01 (-0.02, 0.05) | 0.47 |
| Follow-up time * Age | 0.004 (0.001, 0.007) | 0.002 |
| Black race (ref. White/other) | 2.09 (-0.54, 4.73) | 0.13 |
| Follow-up time * Black race | -0.07(-0.28, 0.14) | 0.52 |
| RAAS blockade use | 1.76 (0.8, 2.72) | <0.001 |
| Diabetes | 1.3 (0.36, 2.24) | 0.007 |

Figure S1. Annual eGFR decline pre- and post-AVF creation over different observational periods

Figure S1 displays the eGFR in mL/min/1.73 m^2^, calculated using the CKD-EPI formula, against the time in days before and after AVF creation, the latter represented by time 0. The estimates are represented by the bold solid line with the 95% confidence interval in dotted lines. The eGFR decline after AVF creation was slowed by 1.28 mL /min/1.73 m^2^ in the 6 months (A), by 0.78 mL/min/1.73 m^2^ in the 12 months (B) and by 1.34 mL/min/1.73 m^2^ in the 18 months (C), pre- and post AVF creation.
